# A Novel Pavement Abrasion Test for Assessing Injury Risk to Vulnerable Road Users

**DOI:** 10.3390/s25206275

**Published:** 2025-10-10

**Authors:** David Llopis-Castelló, Carlos Alonso-Troyano, Pablo Álvarez-Troncoso, Aida Marzá-Beltrán, Alfredo García

**Affiliations:** 1 Highway Engineering Research Group, Universitat Politècnica de València, Camino de Vera s/n, 46022 Valencia, Spain; caraltro@upv.es (C.A.-T.); agarciag@tra.upv.es (A.G.); 2 BECSA S.A.U., Edificio Simetría, Paseo Buenavista s/n, 12100 Castellón, Spain; palvarez@becsa.es (P.Á.-T.); amarza@becsa.es (A.M.-B.)

**Keywords:** pavement, surface abrasion, micromobility safety, non-destructive testing, surface characterization, injury risk assessment

## Abstract

**Highlights:**

**What are the main findings?**

- A novel abrasion test was developed to quantify pavement aggressiveness using paraffin wax as a skin proxy.
- The method demonstrated high sensitivity and repeatability across different pavement materials.

**What is the implication of the main finding?**

- The test enables injury-risk assessment for vulnerable road users beyond traditional friction-based safety metrics.
- It provides a new criterion for safer pavement design by incorporating post-impact performance into infrastructure evaluation.

**Abstract:**

This study introduces a novel and user-centered surface abrasion test designed to assess the injury potential of pavement surfaces, particularly for vulnerable road users such as micromobility users. Traditional pavement evaluation methods focus on skid resistance and texture but do not account for the surface’s mechanical aggressiveness during a fall. To address this gap, the proposed test simulates fall conditions by dragging a paraffin wax specimen—used as a low-cost and reproducible proxy to approximate the abrasive response that could affect human skin—over pavement at a controlled speed and load, quantifying material loss as an indicator of surface abrasiveness. The method was validated on three pavement types (smooth ceramic, bituminous, and concrete), demonstrating its sensitivity and repeatability. Unlike conventional point-based tests, it enables continuous evaluation along a predefined length, offering more representative results. A full-scale case study on a micromobility-dedicated bike lane confirmed the test’s responsiveness to surface changes over time. Results suggest the method is practical, reproducible, and applicable to a wide range of pavements. Beyond micromobility, it can be extended to other vulnerable users, such as motorcyclists. The test represents a new metric for infrastructure safety audits focused on injury mitigation.

## 1. Introduction

Over the past decade, the transportation sector has undergone a profound transformation driven by environmental concerns, urban congestion, and the growing need for sustainable mobility alternatives. Micromobility—characterized by the use of lightweight personal mobility vehicles (PMVs) such as bicycles, electric scooters, and e-bikes—has emerged as a key response to these challenges. Encouraged by Sustainable Urban Mobility Plans (SUMPs) [1,2,3], this mode of transport offers significant advantages for short-distance trips and last-mile connectivity, especially in densely populated urban environments [4,5].

The adoption of micromobility has accelerated in recent years due to external factors such as the COVID-19 pandemic and rising fuel costs. Between 2020 and 2024, PMV use increased by approximately 40% [6], underscoring a shift in urban travel preferences. This emerging mode of transport has demonstrated clear benefits for urban safety and accessibility. In densely populated urban areas, the likelihood of fatal accidents is considerably higher for motor vehicles than for PMVs [7]. Nevertheless, micromobility also introduces new safety risks for users and pedestrians, most of which are closely linked to the design and planning of dedicated infrastructure, such as bike lanes.

According to the European Transport Safety Council [8], 53% of all cyclist fatalities in the European Union are the result of collisions with passenger cars, while 16% are caused by single-bicycle crashes (SBCs). This latter figure exceeds the proportion of fatalities involving heavy vehicles (13%), vans (7%), buses (2%), and other vehicles (6%). These statistics highlight the growing relevance of single-user incidents—especially those involving falls without collision—in the overall safety landscape of micromobility.

Such incidents often result in users falling directly onto the pavement surface, making the physical properties of the pavement a key factor in injury severity. While traditional safety studies have focused on skid resistance, which reduces the likelihood of accidents, and vibration exposure, which affects comfort [9,10,11], these parameters do not account for the potential harm caused upon impact. Among the physical properties of pavement, surface abrasion plays a particularly critical role, as it can determine the extent of skin injury or trauma when a user’s body slides over the pavement. Despite its relevance, abrasion potential is rarely considered in infrastructure diagnostics or standards. However, recent works have emphasized the importance of surface texture and wear mechanisms—albeit from the perspective of tire–pavement interaction—reinforcing the relevance of surface degradation as a safety-related issue [12].

Research in micromobility infrastructure typically groups the factors influencing safety and performance into four main pillars, in accordance with guidelines from the National Association of City Transportation Officials (NACTO) [13]: geometry, pavement, traffic, and operational conditions. Among these, pavement has received increasing attention. Studies have shown that skid resistance is significantly lower on smooth or painted tile surfaces compared to asphalt or concrete [9] and that concrete pavements generate higher vibrations for e-scooter users than Hot Mix Asphalt (HMA) [10]. Mobile sensing approaches have even been used to monitor surface conditions and comfort indicators via smartphones [11].

Recent advances in the evaluation of pavement surface performance have focused extensively on skid resistance and surface texture analysis, particularly under abrasion-induced degradation. Qian et al. [14] proposed a detailed methodology to investigate how surface abrasion affects the anti-skid performance of asphalt pavements, introducing a combined use of high-precision laser scanning and fractal analysis to characterize macro- and micro-texture degradation and correlate it with skid resistance. In a similar context, other authors have also explored friction modelling using laser-based texture measurements [15,16,17,18] and fractal-based surface descriptors [19,20,21], reinforcing the relevance of multiscale surface evaluation in pavement safety research. Their work highlights how surface evolution due to traffic-induced aggregate polishing affects adhesion and hysteresis mechanisms at the tire–pavement interface, which are in turn influenced by surface roughness at different scales [22,23]. Key indicators such as Mean Profile Depth (MPD), Mean Texture Depth (MTD), and fractal dimension D were shown to correlate strongly with British Pendulum Number (BPN) and other friction coefficients [24,25,26], allowing for predictive modelling of skid resistance in road safety assessments. This is consistent with the three-phase friction evolution described by Kokkalis [27], where an initial bituminous film reduces skid resistance, followed by a rapid increase due to the exposure of aggregate microtexture and a subsequent polishing phase leading to gradual friction reduction. This trend has also been observed by Miao et al. [28], who documented logarithmic friction loss patterns over time, particularly in the case of dense and rubber asphalt concrete.

However, as thorough and technologically advanced as these approaches are, their primary focus remains on vehicle–road interaction, especially under service-induced abrasion of the pavement material itself [29]. While valuable for understanding pavement durability and vehicle control, these methods do not address an equally critical safety dimension: the impact of pavement surface aggressiveness on human skin during a fall. In contrast, the present study aims to fill this gap by introducing an abrasion-based testing method focused on user injury risk, particularly relevant for vulnerable micromobility users involved in single-vehicle crashes. The key innovation lies in shifting from tire-centred metrics to user-centred metrics of pavement aggressiveness. This perspective is novel and complementary to existing surface performance evaluations, enriching the broader discussion on pavement safety by integrating a user-centred, injury-oriented metric into the assessment framework. This complements recent durability-focused reviews such as that of Al-Taher et al. [30], which, while emphasizing material wear from mechanical and environmental stressors, highlight the need for new testing approaches that can simulate surface-level deterioration in safety-relevant contexts.

In addition to traditional approaches, recent studies have emphasized the complexity of the frictional mechanisms involved at the pavement–user interface. Friction is not only a function of material type and surface texture but also a result of micro- and macro-scale interactions governed by pavement texture characteristics across multiple wavelengths and amplitudes [22,31]. Micro-texture is critical for low-speed friction, while macro-texture plays a dominant role at higher speeds, particularly affecting water drainage and hydroplaning behaviour [31,32]. Despite efforts to link texture indices with measured friction coefficients, many studies report inconsistent correlations, suggesting that texture–friction relationships are influenced by multiple, interdependent factors such as speed, load, or surface contaminants [33,34].

The development of advanced texture measurement techniques, including 3D laser scanning and digital image-based methods, has helped deepen the understanding of pavement topography and its relationship with skid resistance [35,36,37]. These approaches allow for detailed spatial analysis of surface irregularities, going beyond traditional profile-based parameters like MPD or MTD. Nonetheless, even with improved texture descriptors, existing methods remain focused on vehicle-pavement interaction—whereas this study uniquely addresses the abrasion effects experienced by human skin in fall scenarios. This shift in perspective, from tire friction to user injury risk, represents a novel contribution to pavement safety research.

Although several test methods exist for assessing pavement surface characteristics, none are specifically designed to quantify abrasion-related injury risk in micromobility contexts. The most widely used surface evaluation techniques include the British Pendulum Tester (BPT) (UNE-EN 13036-4:2012) [38] and the Dynamic Friction Tester (DFT) (ASTM E1911) [39], both of which measure surface friction to assess skid resistance. These tools are widely adopted in road safety evaluations due to their proven reliability for identifying accident-prone conditions. However, they focus solely on frictional performance, without considering the abrasive potential of surfaces in the event of a fall, thus overlooking a critical component of injury severity for vulnerable road users like cyclists or users of e-scooters.

In parallel, industries such as protective textiles and materials testing have developed standardized methods to evaluate abrasion resistance, including the Martindale Abrasion Test (UNE-EN ISO 12947-2:2016) [40] and the Taber Abrasion Test (ASTM D3884) [41]. These tests involve subjecting a sample material to repeated mechanical rubbing using standard abrasive surfaces under defined pressures and cycles. Although effective for comparing wear resistance in fabrics, leathers, and coatings, these methods are not directly applicable to pavements, as they do not simulate the dynamics of a body sliding across a hard surface. The materials, contact pressures, and kinematics involved are fundamentally different from those present in micromobility-related falls. To the authors’ knowledge, no standardized procedure exists for evaluating pavement surface abrasion in relation to injury potential. This absence represents a critical research gap in pavement design and safety assessment, particularly as cities invest in safer micromobility infrastructure. This is particularly relevant as current infrastructure safety frameworks lack threshold values or regulatory criteria linking surface abrasion with injury risk—something urgently needed for both micromobility infrastructure and roads.

In the context of pavement surface degradation, abrasion is a critical phenomenon associated with the relative movement between vehicle tires and the road surface. While friction has been extensively studied from the perspective of safety and control, abrasion is more directly linked to mechanical deterioration of the surface, affecting long-term performance and injury risk for vulnerable users. In rigid pavements, abrasion occurs as a result of impact, grinding, and local crushing due to dynamic loading, with rates ranging from 0.04 to 0.5 mm per year depending on traffic intensity and environmental conditions [42].

Abrasion resistance is not a bulk property like compressive or flexural strength; rather, it is governed by surface characteristics and is sensitive to factors such as surface finishing, curing methods, aggregate hardness, and environmental exposure [43,44]. Studies have shown that the abrasion process in concrete pavements often leads to mortar loss, aggregate loosening, dust generation, and eventually reduced surface skid resistance, particularly in areas subjected to studded tires or debris impact [45]. These observations further support the need for test methods that simulate surface-level deterioration mechanisms and their consequences, especially under real-world conditions.

However, despite the recognition of abrasion as a key surface-level failure mode in both flexible and rigid pavements, standardized procedures to evaluate its effects on user safety—especially for non-motorized or unprotected users—are still lacking.

To address this gap, the present study proposes a novel surface characterization test designed to simulate and quantify surface-induced abrasion under realistic conditions. The method consists of dragging a paraffin wax specimen—selected for its physical and mechanical similarity to human skin—across a pavement surface at a constant speed and under a known load. The resulting mass loss is used as an objective and reproducible metric of surface aggressiveness. Unlike point-based friction measurements, this test captures surface behaviour continuously along a predefined length, offering a more representative simulation of real-world fall scenarios.

This approach provides a practical and safety-oriented tool for pavement assessment, with potential applications in infrastructure design, material selection, and accident severity analysis for vulnerable road users. Its incorporation into safety auditing protocols could help inform new design standards that prioritize both slip prevention and injury mitigation.

## 2. Method

This study aimed to develop and validate a novel test method for quantifying the abrasion potential of pavement surfaces, with a focus on evaluating the severity of injuries that vulnerable road users may sustain during falls. The methodology was structured in four main stages: material selection, device and procedure development, test validation, and real-world application through a case study.

The first step involved identifying a test material capable of reliably simulating the mechanical response of human skin under abrasive conditions, serving as a physical proxy to estimate the relative aggressiveness of pavement surfaces toward human skin. To guide the selection process, a set of technical, practical, and safety-based criteria was established, followed by a comprehensive market survey to identify suitable candidates. Once selected, the material was integrated into the design of a novel abrasion testing device, specifically conceived to replicate the dynamics of a fall.

The testing apparatus was prototyped and calibrated to perform controlled trials in which the selected material is dragged across various pavement types at a constant speed and under a known load. The methodology, device, and procedure are described in detail in Section 3. To verify the robustness of the method, a series of validation tests were conducted on different pavement surfaces, distinctly characterized by their texture and composition. This phase was critical to assess the method’s ability to detect significant differences in surface aggressiveness.

Finally, the proposed methodology was applied to a full-scale experimental test section located in Burriana, Spain, allowing the performance of the pavement to be monitored over time through repeated measurements of macrotexture, skid resistance, and abrasion. This case study, detailed in Section 4, provided additional insight into the applicability and consistency of the test under real-life conditions.

## 3. Development of the Abrasion Test Method

### 3.1. Selection of the Test Material

To select an appropriate material for assessing pavement abrasion, a set of fundamental requirements was established. The selected material must:

- Have well-known and stable mechanical properties: Its composition and basic physical characteristics must be clearly defined and consistent.
- Be quantifiable: The mass loss resulting from the material being dragged over the pavement surface must be measurable with a conventional balance.
- Be readily available: The material should be commercially available and well-established in the market, preferably with applications in other industries to ensure future accessibility.
- Be low-cost: Affordability is essential to minimize testing expenses and encourage repeatability across studies.
- Be mouldable: The material must be easy to shape and reproduce, allowing for consistent test specimen dimensions.
- Be non-hazardous: Under normal use and in its original form, the material should pose no health or environmental risks.

Based on these criteria, paraffin wax was selected as the most suitable material for the development of the abrasion test. Paraffin is a petroleum-derived wax that is solid at room temperature and widely used in various industries, including candle production, food processing (e.g., cheese coatings), textile manufacturing (e.g., sportswear), and the cosmetic and pharmaceutical sectors. It is an inert, waterproof, glossy, biodegradable material that combusts cleanly, without releasing harmful or corrosive vapours.

**Figure 1:**
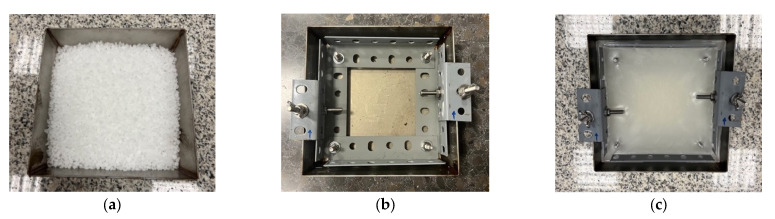
Paraffin wax: (**a**) granular form, (**b**) melted wax, and (**c**) hardened wax.

The paraffin wax used in this study was acquired in granular form (Figure 1a), which is a common commercial presentation for industrial and laboratory uses. This material was selected due to its availability, consistency, and ease of handling. For specimen preparation, the paraffin was melted in a preheated oven at 100 °C and poured into a standardized mould (170 × 170 × 25 mm). Given its melting point of approximately 67.5 °C (as shown in Table 1), the paraffin melts quickly under these conditions (Figure 1b). Once fully liquefied, the mould is removed from the oven and allowed to cool at ambient temperature until solidified (Figure 1c). The cooling period typically lasts less than one hour. Since these thermal steps do not influence the mechanical performance of the specimen, no strict timing is required, which contributes to the practicality and reproducibility of the test.

### 3.2. Design of the Testing Apparatus

To simulate the abrasion process resulting from a fall onto the pavement, this study proposed quantifying the material loss that occurred as a paraffin wax specimen was dragged across a defined length of pavement at a constant speed while subjected to a known load.

**Figure 2:**
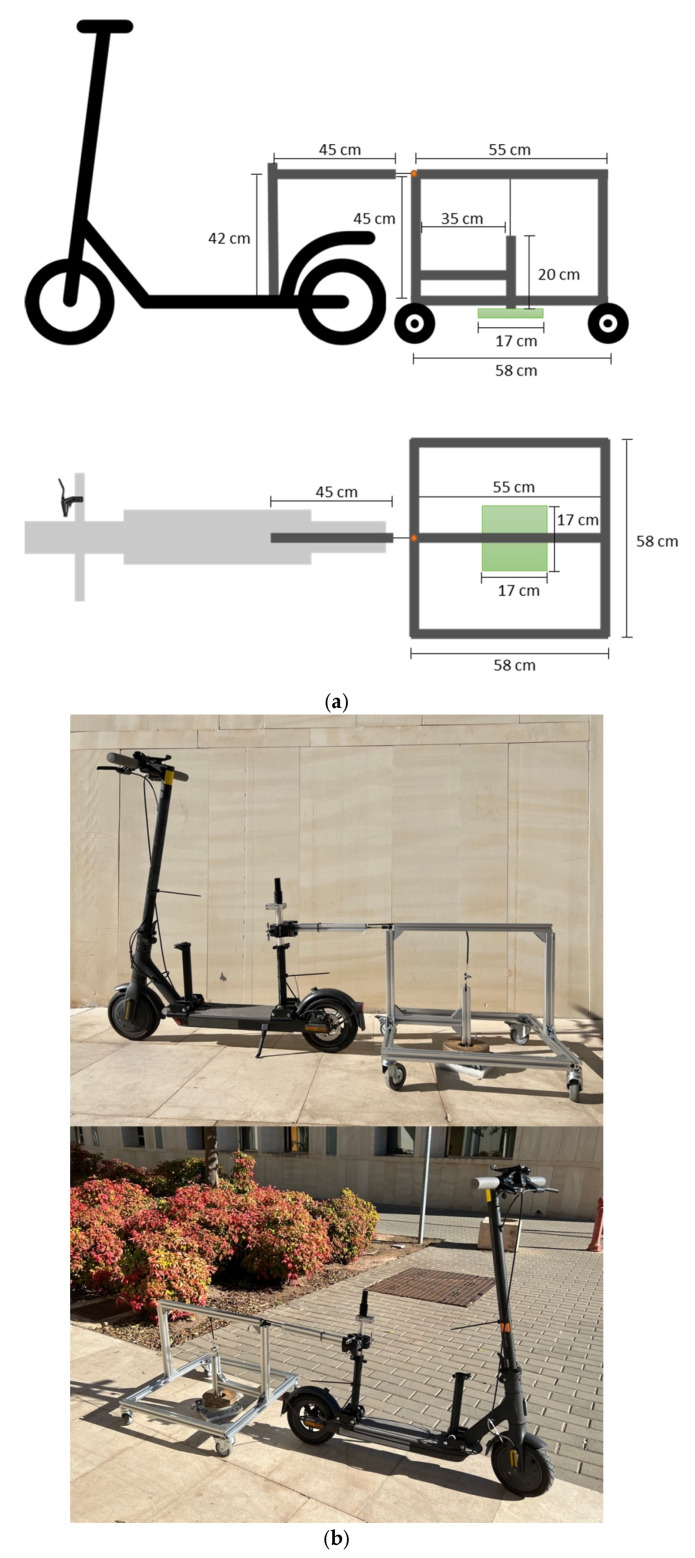
Apparatus for the development of abrasion test: (**a**) design and (**b**) assembly.

To carry out this procedure, a prototype was developed comprising two main components: (i) a towed frame, constructed from metal profiles, which houses the test specimen, and (ii) a control system that enables the precise initiation and termination of the abrasion process. Figure 2 illustrates the structural layout and main components of the device.

The structural frame consists of a metal beam that connects the test vehicle—an electric scooter in this case—to the metal structure containing the specimen. The connection between the two components is made via a ball joint, which allows rotational movement in the horizontal plane while preventing vertical displacement. The specimen-holding structure includes:

- Four base metal profiles equipped with wheels that rest directly on the pavement surface;
- A vertical profile that connects to the scooter via the ball joint and, together with two additional profiles, forms a vertical support frame;
- Two guide rails that enable the vertical movement of the test specimen and load assembly.

The abrasion process is initiated and terminated using a system that simulate the function of a bicycle brake. When the brake is engaged and the cable is tensioned, the test specimen remains lifted off the pavement. When the brake is released, the cable slackens, allowing the specimen to come into contact with the surface. This mechanism consists of the brake lever itself, a vertical metal profile for guiding the up-and-down movement, and a load attachment system designed for quick installation. The specimen is fastened to the testing frame via a bolted connection.

It should also be noted that, to ensure consistent contact between the paraffin specimen and the pavement surface during the test, a ballast of 3.5 kg was added to the test frame. This load was found sufficient to maintain continuous contact without exerting excessive pressure on the paraffin, which could artificially accelerate material loss. The main objective of this load was to simulate realistic sliding contact rather than compressive stress. While heavier loads would indeed increase abrasion, as observed during preliminary trials, this study employed a standardized load for all tests.

Table 2 provides the technical specifications of each system component and indicates the required quantity of each element.

Additionally, to secure the wax specimen to the device, a framework of metal profiles is embedded into the paraffin during the cooling process. This internal structure, which acts as the skeleton of the specimen, facilitates its bolted attachment to the test device (see Figure 1).

### 3.3. Experimental Procedure

The primary objective of the proposed abrasion test is to quantitatively assess the material loss experienced by the paraffin wax specimen when dragged over the pavement surface at a controlled speed and over a defined distance.

The test procedure consists of the following steps:

1. Preparation of the paraffin wax specimen with dimensions of 170 × 170 × 25 mm.
2. Measurement of the initial mass of the specimen prior to testing (*m*_0_).
3. Mounting of the specimen onto the testing apparatus attached to the electric scooter.
4. Sweeping and cleaning of the pavement surface to eliminate debris that could affect the results.
5. Measurement of pavement temperature (*T_p_*) at the testing site.
6. Acceleration of the scooter to reach the target testing speed (*v_a_*) on the pavement section.
7. Dragging of the paraffin wax specimen over the predefined testing length (*l_a_*).
8. Deceleration and stopping of the scooter once the testing distance has been completed.
9. Removal of the specimen from the testing apparatus.
10. Measurement of the final mass of the specimen after testing (*m*_1_).
11. Calculation of abrasion loss by determining the mass difference before and after the test (*m_a_ = m*_0_  *− m*_1_).

This approach enables the quantification of the abrasive effect of different pavement surfaces, offering a replicable and objective method for evaluating pavement aggressiveness in micromobility infrastructure.

### 3.4. Validation

To evaluate the reliability and repeatability of the proposed abrasion test, a series of experiments were conducted on three different pavement types: smooth ceramic pavement, bituminous pavement, and concrete pavement. These surfaces were selected because they represent the most commonly used pavement types in micromobility infrastructure in Spain, particularly in urban environments. While ceramic pavements are still prevalent in older pedestrian areas, bituminous and concrete surfaces have become the preferred options in newly built or rehabilitated bike lanes due to their improved comfort and reduced vibration levels. Moreover, these pavements were selected to ensure that the test could effectively differentiate between surface materials with clearly distinct textural and mechanical properties.

All tests were conducted on the same day so as to minimize the influence of environmental factors—particularly pavement temperature—on the test results. During testing, pavement surface temperatures ranged between 25 °C and 30 °C. Two test lengths—25 m and 50 m—were analysed at a constant speed of 15 km/h. This speed was selected based on previous field studies, which indicated that micromobility users such as cyclists and e-scooter riders typically travel at speeds between 15 and 20 km/h under urban conditions [9]. Therefore, 15 km/h was considered representative of real-world user behaviour while ensuring stability and control during the experimental procedure.

Additionally, the influence of the dragging length on the abrasion process was examined, with the goal of identifying an optimal test length—i.e., one that yields consistent and meaningful abrasion values. For each pavement type and test length, six repetitions were performed, with the first trial discarded in each case as a conditioning run to allow the system to stabilize. After each test, the surface of the paraffin wax specimen was gently cleaned using a soft-bristle brush to avoid contaminating subsequent results.

**Figure 3:**
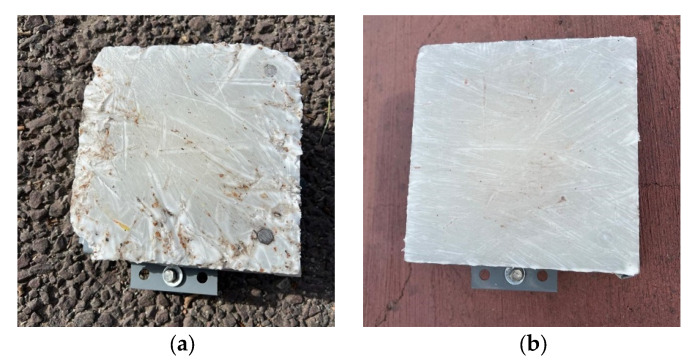
Condition of specimen after test: (**a**) asphalt pavement and (**b**) concrete pavement.

Table 3 presents the measured abrasion values (*m_a_*) recorded during the test campaign. In terms of surface aggressiveness, the concrete pavement exhibited the highest abrasion levels, while the smooth ceramic pavement caused minimal mass loss in the wax specimen. The bituminous pavement displayed abrasion values close to those of the concrete pavement. This outcome is primarily attributed to the noticeable deterioration and presence of surface debris on the bituminous pavement, which led to greater variability in the results, particularly in the 50 m test series (Figure 3).

From the results of these initial tests, it was also observed that the average abrasion is proportional to the dragging length. Specifically, doubling the test length resulted in a twofold increase in mean mass loss for both the bituminous and concrete pavements. This behaviour is consistent with expectations, as doubling the dragging distance is effectively equivalent to performing the shorter test twice under similar conditions. However, this linear relationship did not hold for the smooth ceramic pavement, where the paraffin mass loss remained negligible even when the test length was increased. This outcome is due to the extremely low roughness of the ceramic surface, which generates very limited abrasive interaction regardless of distance. In such cases, the results at different lengths are not directly comparable to those obtained on more abrasive surfaces.

An important advantage of using a longer dragging length—excluding the values obtained on the bituminous pavement due to its contamination and deteriorated state—is a significant reduction in the coefficient of variation, leading to more consistent and stable results. Therefore, it can be concluded, at least preliminarily, that the proposed testing method is suitable for reliably evaluating the abrasion characteristics of pavement surfaces.

## 4. Field Application: Experimental Case Study

Following the initial development and verification of the abrasion test method, a full-scale field case study was conducted to assess the applicability and consistency of the procedure under real-world conditions. To this end, a purpose-built test section was monitored over time, focusing on three key surface performance indicators: macrotexture, skid resistance, and abrasion. This case study not only demonstrates the practical deployment of the test in an urban setting but also provides valuable insights into how these surface characteristics evolve and interact throughout the early service life of the pavement. Ultimately, the results support the use of the method as a reliable tool for monitoring safety-related surface conditions in micromobility corridors.

**Figure 4:**
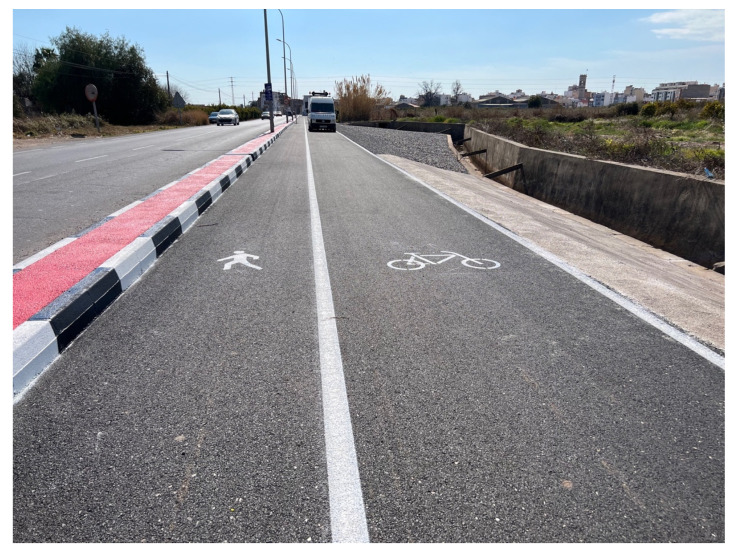
Experimental test section.

The field study was carried out on a newly constructed bike lane located in Burriana (Castellón, Spain). The lane is a segregated path specifically designed for micromobility use, with a total length of 300 m and a width of 3 m. The pavement structure comprises a 15 cm granular subbase and a 4 cm thick Asphalt Concrete (AC16 surf 50/70 S) wearing course. Notably, the surface layer incorporates a sustainable asphalt mixture of 50% Reclaimed Asphalt Pavement (RAP) and 50% ceramic aggregates (see Figure 4), offering a real-world context for evaluating material performance and surface behaviour over time.

**Figure 5:**
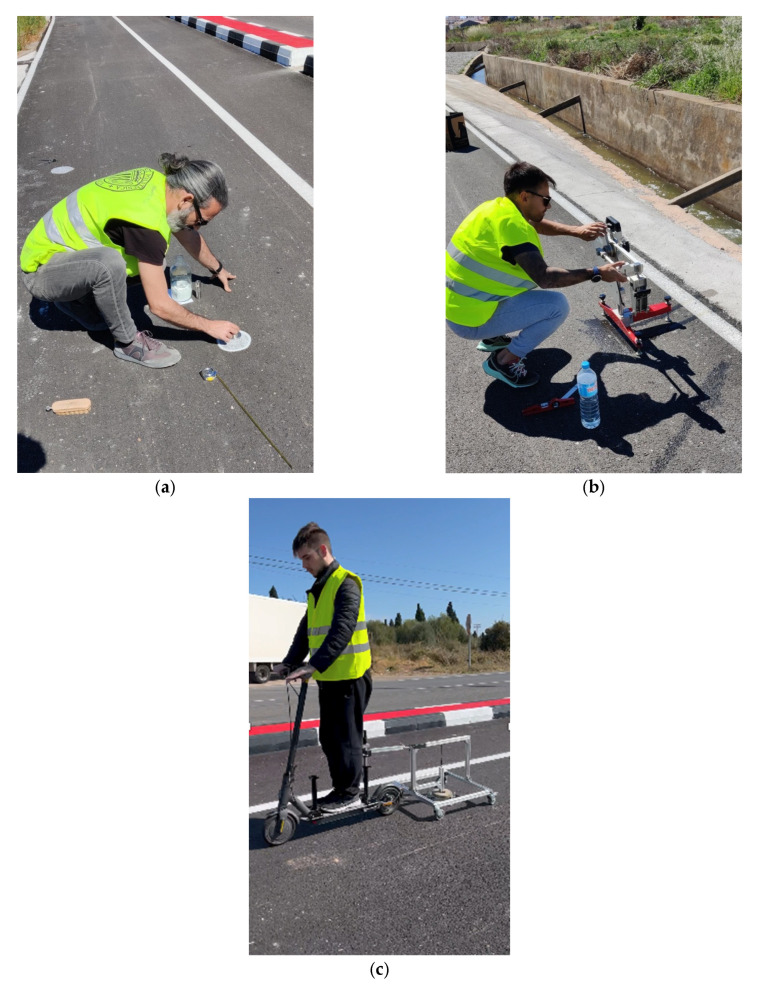
Surface performance analysis: (**a**) macrotexture, (**b**) skid resistance, and (**c**) abrasion.

To assess the surface performance of the test section over time, a monitoring campaign was conducted focusing on three key surface properties: macrotexture, skid resistance, and abrasion. Macrotexture was measured in accordance with UNE-EN 13036-1 (Figure 5a), which provides a geometric characterization of surface roughness using volumetric methods. Skid resistance was assessed using the British Pendulum Tester according to UNE-EN 13036-4 (Figure 5b), which quantifies the frictional behaviour of the pavement. Abrasion was evaluated using the novel test method developed in this study (Figure 5c), which simulates a fall by measuring the mass loss of a paraffin wax specimen dragged across the pavement. These three properties were each measured during three separate campaigns to track surface evolution over time. All tests were performed under dry pavement conditions, with surface temperatures ranging between 25 °C and 30 °C.

**Figure 6:**
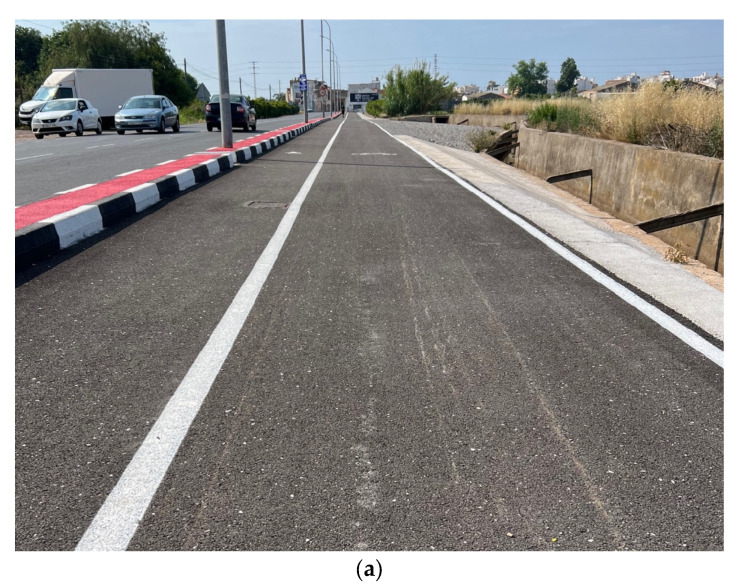

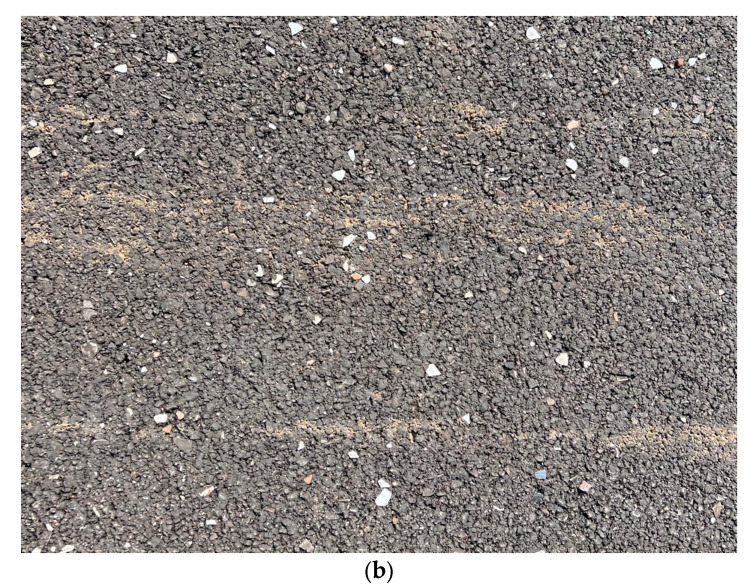
Pavement condition on 2 June 2023: (**a**) general view, (**b**) surface detail.

Macrotexture measurements were conducted following UNE-EN 13036-1. In each campaign, four observations were made (see Table 4). The Mean Texture Depth (MTD) remained relatively stable during the first two measurements (approx. 0.80), with values consistently above the 0.70 mm minimum specified by Spanish technical guidelines (PG-3). However, a significant reduction was noted during the third measurement round (average MTD of 0.69), likely due to the disappearance of the bitumen film and the exposure of glazed ceramic aggregate surfaces (Figure 6). This evolution may have been accelerated by rain events preceding the third campaign.

**Figure 7:**
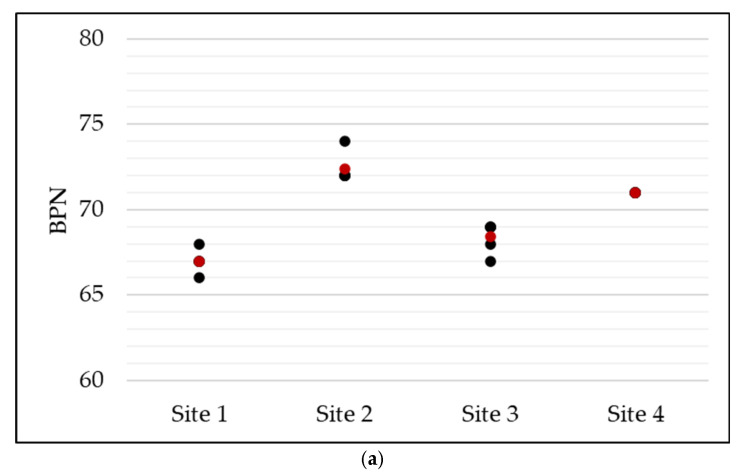

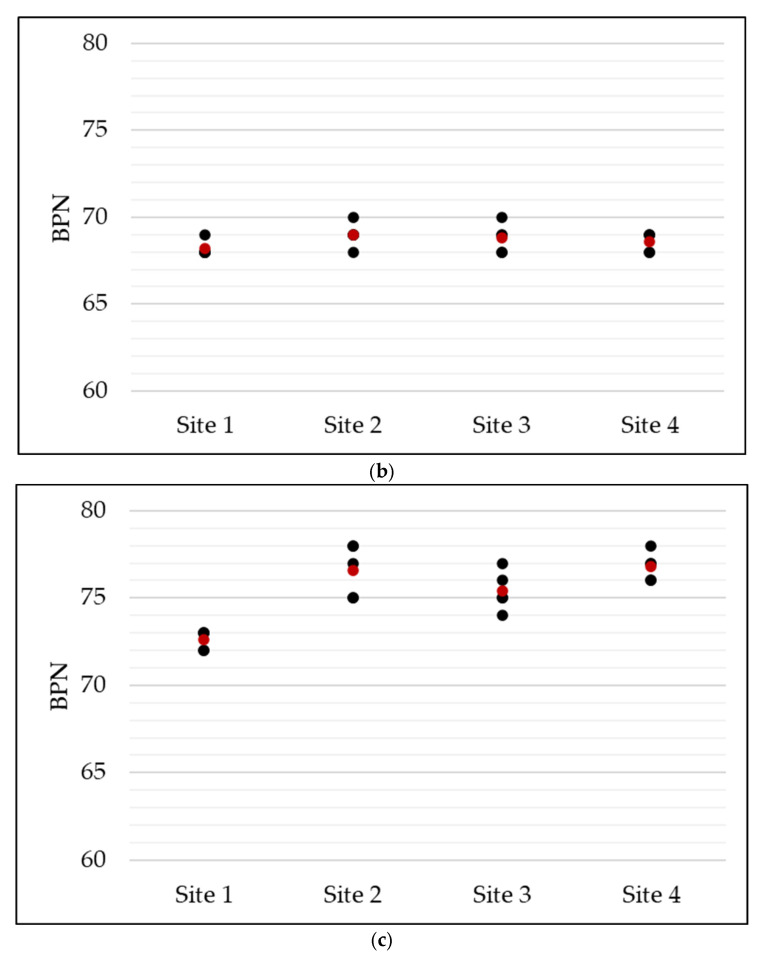
Skid resistance observations according to UNE-EN 13036-4: (**a**) 3 March (**b**) 3 April (**c**) 2 June 2023.

Skid resistance was measured at four locations per campaign following UNE-EN 13036-4. At each point, five readings were taken to identify skid resistance as the average of the British Pendulum Number (BPN). BPN ranged from 67.0 to 72.5 on 3 March 68.0 to 73.0 on 3 April and 72.5 to 77.0 on 2 June (Figure 7a–c). The skid resistance remained relatively stable in the first two campaigns and increased significantly in the third. This improvement is attributed to the removal of the bitumen film by rainfall events in May, which exposed the underlying aggregates and improved surface friction.

This is in line with the common behaviour of friction in asphalt pavements. As traffic and environmental actions remove this film, aggregate microtexture is exposed, leading to an increase in skid resistance until a polishing phase begins.

In this case, despite the presence of glazed ceramic aggregate faces that could potentially reduce roughness, the surface showed improved friction performance, likely due to the initial binder film loss rather than texture degradation.

Abrasion was assessed using the procedure described in Section 3. A paraffin wax specimen was dragged over the surface at 15 km/h over a length of 50 m (Table 5). Five measurements were performed for each distance and campaign.

Abrasion values decreased in the second measurement compared to the first. However, abrasion increased slightly in the third campaign. This is likely due to surface roughening following rainfall, which removed the binder film, consistent with the increase observed in skid resistance. Additionally, the variation in abrasion levels may also be influenced by pavement temperature. During testing, surface temperatures were recorded at 30 °C in March, 39 °C in April, and 35 °C in June. While this suggests a possible correlation between temperature and abrasion behaviour, a more detailed study is needed to determine the extent and mechanisms through which temperature affects the outcome of the test.

The combination of macrotexture, skid resistance, and abrasion assessments offers a comprehensive understanding of the surface performance of micromobility infrastructure. Macrotexture, quantified through volumetric methods, serves as a geometric indicator of surface roughness and plays a fundamental role in water drainage and tire-pavement interaction. Specifically, macrotexture encompasses surface irregularities with wavelengths ranging from 0.5 to 50 mm, which are large enough to affect friction at high speeds, surface runoff, and splash generation. Skid resistance, derived from dynamic friction testing, directly reflects the pavement’s ability to prevent loss of control, especially in wet conditions, and is essential for accident prevention. Abrasion, as measured by the novel method developed in this study, introduces an injury-oriented perspective by quantifying the pavement’s potential to cause harm in the event of a fall—a risk particularly relevant to micromobility users. Together, these parameters represent a multi-dimensional evaluation: macrotexture supports functional performance, skid resistance ensures operational safety, and abrasion provides critical insight into post-incident severity. Their combined analysis not only strengthens infrastructure diagnosis but also helps guide the design of pavements that are safe, durable, and user-centered.

## 5. Discussion

The results of this study confirm the relevance and potential of the proposed abrasion test method as a valuable complement to existing surface performance assessments for pavements. Unlike conventional methods that focus primarily on skid resistance or surface texture deterioration due to traffic-induced polishing [46,47], the test introduced here provides a direct estimation of surface aggressiveness toward the human body. This is particularly critical in micromobility contexts, where single-user falls are common and the pavement’s abrasiveness can significantly influence injury severity.

Compared to established standards such as the British Pendulum Test (UNE-EN 13036-4) or the Dynamic Friction Tester (ASTM E1911), which quantify the risk of slipping, or surface scanning techniques used for texture analysis [13,14,15,16,17,18,19,20,21,22,23,24], the proposed method addresses a previously unquantified dimension of user safety. While the above techniques focus on fall prevention, this method supports injury mitigation by characterizing the abrasiveness that a user would experience once a fall has occurred. This distinction is crucial in moving from a friction-centred paradigm to a user-centred approach to pavement evaluation.

A particularly important methodological distinction is that, unlike conventional skid resistance tests—which are point-based and provide friction values at isolated spots—the abrasion test developed in this study is a continuous test. By measuring surface aggressiveness along a predefined path, it captures the spatial variability of pavement behaviour and identifies localized defects that may otherwise go undetected. This continuous approach enhances diagnostic resolution and reflects more accurately the dynamic nature of falls experienced by vulnerable road users.

In recent years, studies such as those by Qian et al. [14], Ding et al. [16], and Miao et al. [19] have advanced the understanding of pavement friction by introducing high-resolution surface profiling, spectral analysis, and fractal modelling. However, these efforts primarily address the deterioration of pavement texture and its impact on tire–road interaction. Likewise, the recent literature has explored the influence of micro- and macro-texture on skid resistance under different operational conditions [26,30,34], and others have investigated the use of digital image analysis or laser scanning for refined texture measurement [31,32,33]. Still, none of these methods evaluate the mechanical interaction between a falling body and the pavement surface itself. The proposed test fills this methodological gap by simulating a realistic fall scenario, thus introducing a new dimension to surface performance analysis: the injury potential.

The validation tests, conducted on ceramic, bituminous, and concrete surfaces, demonstrated the method’s reproducibility, sensitivity, and internal consistency. The abrasion values obtained were logically consistent across repetitions and proportional to the dragging length. Moreover, in the case study, the test proved applicable in real-world conditions and responsive to surface changes over time—notably capturing the impact of rainfall-induced texture changes, which also corresponded to increases in measured skid resistance. This reinforces the method’s ability to reflect surface evolution in active environments. Such responsiveness supports the potential integration of the abrasion index into pavement condition monitoring systems.

An additional strength of the method is its operational simplicity and low implementation cost, particularly when compared to high-end surface scanning or sensor-based systems. These qualities make it suitable not only for experimental research but also for on-site diagnostics in municipal or regional pavement evaluations.

While this research has focused mainly on micromobility infrastructure, the method could be extended to other road users, such as motorcyclists, who are also vulnerable to injuries in sliding accidents. For such applications, the abrasion index could serve as a new criterion for evaluating road safety beyond traction and drainage properties, especially in curves, transition zones, or intersections. It may also prove useful in evaluating pedestrian crossings, shared-use paths, or accident-prone locations, where impact-related injury risk is elevated.

To enhance the robustness and generalizability of the method, future work should consider:

- Increasing the number of test repetitions per pavement type;
- Testing at different speeds (e.g., 10 and 20 km/h) and with various loads;
- Investigating the effect of pavement temperature on paraffin wax abrasion behaviour.
- Evaluating additional surface materials, including innovative or recycled aggregates, fibre-reinforced pavements, or polymer-modified surfaces.

In parallel, there is a need to define threshold values for surface abrasiveness. These thresholds should be calibrated in relation to acceptable levels of skid resistance, ensuring that pavements maintain a balance between grip and post-impact safety. Establishing a correlation between the abrasion index and skid resistance could guide the development of dual-performance criteria, aligning functional design standards with user protection requirements. Furthermore, collaborations with biomechanics or trauma research could help calibrate the abrasion index with real injury risk thresholds, potentially informing regulatory frameworks.

In summary, this study introduces a novel, reproducible, and cost-effective test method that complements existing surface evaluation tools by targeting a previously overlooked performance parameter: injury potential due to surface abrasion. Its original contribution lies in bridging the gap between friction-based safety metrics and the mechanical consequences of a fall. This has clear implications for infrastructure design, maintenance prioritization, and regulatory development, particularly in the context of increasing micromobility adoption and the broader safety of vulnerable road users.

## 6. Conclusions

This study presents the development, verification, and field application of a novel surface abrasion test designed to assess the injury potential of pavement surfaces, particularly in the context of micromobility infrastructure. Unlike conventional methods that focus on frictional performance or surface durability, the proposed test method provides a unique, injury-oriented perspective by quantifying the material loss of a paraffin wax specimen—a proxy for human skin—dragged across the pavement at a controlled speed and load.

The method proved to be both reproducible and sensitive to different pavement types, successfully differentiating between surfaces such as smooth ceramic, bituminous, and concrete. Furthermore, its continuous nature—measuring abrasion along a defined length—allows for a more representative evaluation of surface behaviour than point-based methods. This continuous measurement strategy enhances spatial resolution and enables the identification of localized surface conditions that may contribute to injury risk. Field application confirmed the test’s practicality under real-world conditions and its responsiveness to surface changes over time, reinforcing its potential as a tool for ongoing infrastructure monitoring.

The originality of this method lies in shifting the focus from vehicle-centred safety indicators (e.g., friction) to human-centred impact analysis. By doing so, it introduces an entirely new dimension to pavement performance evaluation that addresses the consequences of a fall rather than merely its likelihood. Beyond micromobility, the abrasion test holds promise for application in motorized transport environments, such as for assessing the safety of pavements used by motorcyclists. By expanding safety evaluation beyond slip resistance and vibration exposure, this method introduces an additional layer of risk assessment that can inform pavement design, rehabilitation, and material selection.

To advance the method toward standardization and broader implementation, future research should:

- Increase the number of test repetitions per pavement type to strengthen statistical robustness.
- Investigate additional speeds, dragging lengths, and loading configurations.
- Study the impact of environmental variables, such as pavement temperature.
- Define acceptable thresholds for surface abrasiveness in relation to slip resistance, enabling dual-performance safety criteria.
- Explore correlations between the abrasion index and injury severity, potentially linking mechanical test results with biomedical or trauma data.

Overall, the proposed abrasion test contributes a novel and practical approach to pavement safety evaluation, bridging a crucial gap in injury mitigation for vulnerable road users. Its simplicity, adaptability, and focus on post-impact conditions position it as a valuable complement to existing testing protocols in both urban and interurban infrastructure contexts. As cities evolve toward more inclusive and sustainable mobility systems, tools such as this will be essential for ensuring that infrastructure not only prevents accidents but also minimizes their consequences.

Overall, the proposed abrasion test contributes a novel and practical approach to pavement safety evaluation, bridging a crucial gap in injury mitigation for vulnerable road users. Its simplicity, adaptability, and focus on post-impact conditions position it as a valuable complement to existing testing protocols in both urban and interurban infrastructure contexts.

## 7. Patents

The non-destructive test developed in this research for measuring pavement abrasion has resulted in a patent (ES 2990072 B2).

## Figures and Tables

**Table 1:** Properties of paraffin wax. Source: Gran Velada S.L.

Property	ASTM Method	Unit	Min.	Typical	Max.
Congealing point	D-938	°C	65	67	69
Melting point	D-87	°C	65	67.5	70
Oil content	D-721	wt.%	–	0.7	1
Saybolt colour	D-156	–	+22	+30	–
Visual colour	–	–	–	White	–
Needle penetration at 25 °C	D-1321	1/10 mm	–	18	20
Kinematic viscosity at 100 °C	D-445	cSt	6	7	8
Odor	D-1833	–	–	0	1

**Table 2:** Specifications of the elements that make up the abrasion test apparatus.

Item	Manufacturer	Specifications	Quantity
30 × 30 mm metal profiles, slot 8	Alu10	55 cm	5
		45 cm	3
		35 cm	1
		20 cm	1
Bolts	Alu10	M6	24
Brackets	Alu10	M6	12
Wheels	RS Pro	Ø 75 mm	4
Seatpost	SMARTGYRO	–	1
Multi-clamp rack mount	Gibraltar	17.78 × 17.78 × 6.86 cm	1
Rod end joint	Igus	M8	1
Load (ballast weight)	–	3500 g	1

**Table 3:** Abrasion test results.

Type of Pavement	Id	*m_a,_*_25_ (g)	*m_a,_*_50_ (g)
Smooth ceramic pavement			
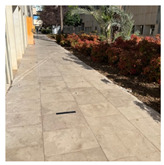	1	0.0	0.2
	2	0.0	0.2
	3	0.1	0.2
	4	0.1	0.0
	5	0.0	0.1
	Mean	0.040	0.140
	Standard Deviation	0.055	0.089
	Coefficient of variation	1.369	0.639
Asphalt pavement			
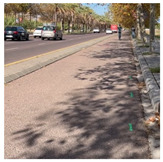	1	3.4	4.8
	2	3.5	7.2
	3	2.5	7.0
	4	2.9	6.5
	5	2.5	4.2
	Mean	2.960	5.940
	Standard Deviation	0.477	1.356
	Coefficient of variation	0.161	0.228
Concrete pavement			
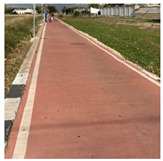	1	5.0	7.4
	2	3.2	6.8
	3	3.7	6.7
	4	4.0	6.9
	5	3.0	7.0
	Mean	3.780	6.960
	Standard Deviation	0.789	0.270
	Coefficient of variation	0.209	0.039

**Table 4:** Macrotexture observations according to UNE-EN 13036-1.

Date	Location	Volume (mm^3^)	Diameter (mm)	MTD (mm)
3 March 2023	1	25,000	199	0.80
	2	25,000	200	0.80
	3	25,000	294	0.85
	4	25,000	209	0.73
3 April 2023	1	25,000	188	0.90
	2	25,000	189	0.89
	3	25,000	210	0.72
	4	25,000	212	0.71
2 June 2023	1	25,000	208	0.73
	2	25,000	220	0.66
	3	25,000	217	0.68
	4	25,000	217	0.68

**Table 5:** Abrasion observations using the proposed test method.

Date	Id	*m_a,_*_50_ (g)
3 March 2023	1	3.8
	2	2.6
	3	3.1
	4	2.4
	5	3.2
	Mean	3.02
	Standard Deviation	0.55
	Coefficient of variation	0.18
3 April 2023	1	2.4
	2	3
	3	1.2
	4	2.8
	5	3.4
	Mean	2.56
	Standard Deviation	0.84
	Coefficient of variation	0.33
2 June 2023	1	5.4
	2	2.6
	3	2.2
	4	2.4
	5	4.2
	Mean	3.36
	Standard Deviation	1.39
	Coefficient of variation	0.41

## Data Availability

The data presented in this study are available from the corresponding author on request. The data are not publicly available due to a Non-Disclosure Agreement.
